# IL-33 induced gene expression in activated Th2 effector cells is dependent on IL-1RL1 haplotype and asthma status

**DOI:** 10.1183/13993003.00005-2024

**Published:** 2024-06-20

**Authors:** Akshaya Keerthi Saikumar Jayalatha, Marlies E. Ketelaar, Laura Hesse, Yusef E. Badi, Nazanin Zounemat-Kermani, Sharon Brouwer, Nicole F. Dijk, Maarten van den Berge, Victor Guryev, Ian Sayers, Judith E. Vonk, Ian M. Adcock, Gerard H. Koppelman, Martijn C. Nawijn

**Affiliations:** 1 University of Groningen, University Medical Center Groningen, GRIAC Research Institute, Department of Pathology and Medical Biology, Groningen, The Netherlands; 2 University of Groningen, University Medical Center Groningen, GRIAC Research Institute, Beatrix Children's Hospital, Department of Pediatric Pulmonology and Pediatric Allergology, Groningen, The Netherlands; 3 National Heart and Lung Institute, Department of Respiratory Cell and Molecular Biology, Imperial College London, London, UK; 4 University of Groningen, University Medical Center Groningen, GRIAC Research Institute, Department of Pulmonary Diseases, Groningen, The Netherlands; 5 University of Groningen, GRIAC Research Institute and European Research Institute for the Biology of Ageing, Groningen, The Netherlands; 6 Centre for Respiratory Research, NIHR Biomedical Research Centre, School of Medicine, Biodiscovery Institute, University of Nottingham, Nottingham, UK; 7 University of Groningen, University Medical Center Groningen, GRIAC Research institute, Department of Epidemiology, Groningen, The Netherlands; 8 Shared first authorship

## Abstract

Asthma is a heterogeneous respiratory disease caused by the interaction between environmental and genetic factors [1]. The *IL-33* and *IL-1RL1* genes are strongly associated with childhood-onset and type-2 high asthma, and the asthma risk alleles amplify interleukin (IL)-33 pathway activity [2]. Environmental factors, such as allergens and viral infections, trigger bronchial epithelial cells to release IL-33, which can activate signalling by binding to the IL-1RL1/IL-1RAcP receptor complex [3], and contribute to hyper-responsiveness, remodelling and chronic type 2 inflammation of the airways [4].

## Shareable abstract

**IL-33 response in Th2 cells is specific to asthma and represents a high risk haplotype, highlighting its role in airway wall cells. Yet, its detection is challenging in bulk asthma transcriptomes due to the scarcity of effector Th2 cells.** https://bit.ly/3WhuMbo

*To the Editor*:

Asthma is a heterogeneous respiratory disease caused by the interaction between environmental and genetic factors [1]. The *IL-33* and *IL-1RL1* genes are strongly associated with childhood-onset and type-2 high asthma, and the asthma risk alleles amplify interleukin (IL)-33 pathway activity [2]. Environmental factors, such as allergens and viral infections, trigger bronchial epithelial cells to release IL-33, which can activate signalling by binding to the IL-1RL1/IL-1RAcP receptor complex [3], and contribute to hyper-responsiveness, remodelling and chronic type 2 inflammation of the airways [4]. *IL-1RL1* is expressed in immune and structural cells of the airways, such as epithelial cells, mast cells, macrophages, Th2 cells and type 2 innate lymphoid cells. *IL-1RL1* encodes two protein isoforms: the transmembrane receptor subunit (IL-1RL1b) and a soluble (IL-1RL1a) isoform that functions as an antagonistic decoy receptor. Human Th2 cells respond to IL-33 by enhancing cytokine production [5]. Genetic variation at the *IL-1RL1* locus, particularly rs1420101 in intron 5 and a block of four non-synonymous single nucleotide polymorphisms (SNPs) in full linkage disequilibrium in exon 11, alter *IL-1RL1* expression levels and IL-33 induced signalling activity [2, 6]. However, it is not known whether genetic variation at the *IL-1RL1* locus affects the response of Th2 cells to IL-33. Therefore, we tested whether *IL-1RL1* haplotype altered the IL-33 induced response of Th2 cells from healthy controls and patients with asthma. Moreover, we explored whether IL-33-induced gene signatures from Th2 cells could identify subgroups of asthma patients in transcriptomic datasets.

We selected peripheral blood mononuclear cells (PBMCs) from asthma patients and controls (figure 1a and b) based on the genotype of asthma-associated *IL-1RL1* SNPs and grouped them into carriers of the high risk haplotype (rs1420101-AA, rs4988956-GG) or low risk haplotype (rs1420101-GG, rs4988956-AA/AG). CD4^+^CD25^−^ T cells were isolated from PBMCs and differentiated into Th2 cells (CellXVivo, #CDK002). Th2 cells were re-stimulated through CD3 and CD28 (555725, BD Pharmingen) in the presence/absence of 100 ng·mL^−1^ IL-33 (rhIL-33, 3625-IL-010, BioTech). RNA was extracted and sequenced using NextSeq500 (Illumina, San Diego, CA, USA), processed data can be found at GEO. Univariant (paired) analysis of differential gene expression induced by CD3/CD28 crosslinking or IL-33 was performed using Limma-voom in R, stratifying by haplotype or disease status of the donor. We generated an IL-33 response signature in Th2 cells by selecting those differentially expressed genes (DEGs) specifically expressed in T cells (using the Human Lung Cell Atlas (HLCA)) by removing genes also expressed in mast cells, macrophages and eosinophils [7].We subsequently selected the genes with top-25% baseline expression to allow detection in single-cell RNA-sequencing data and therein the genes with the top-5% largest LogFoldChange after IL-33 stimulation. Enrichment of this Th2-IL33 gene signature was analysed in single-cell RNA-sequencing data [7] and in bulk RNA-sequencing [8] data from asthma patients and controls in the INDURAIN [9] (bronchial biopsies) and U-BIOPRED [10] (induced sputum) studies using gene set variation analysis [11]. Statistical tests included t-test, Wilcoxon rank-sum test, and Fisher's exact test.

**FIGURE 1:**
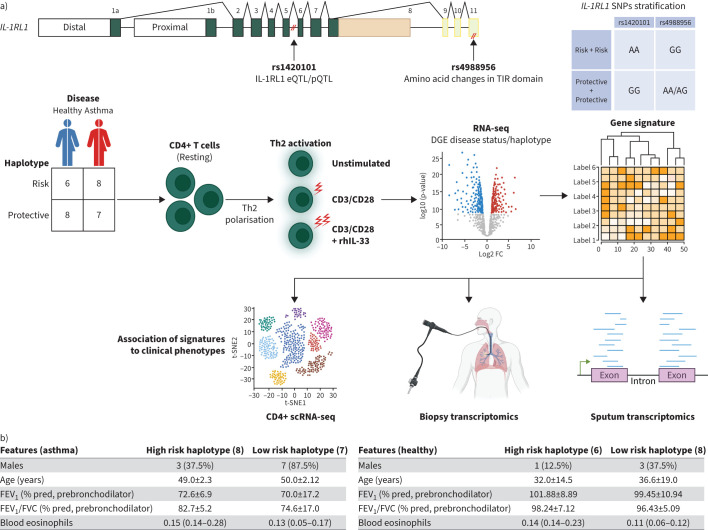
a) Study design: asthma-associated single nucleotide polymorphisms (SNPs) in the *IL-1RL1* gene (rs1420101 and rs4988956) with the selected risk and protective alleles. CD4^+^CD25^−^ T cells from asthma patients and healthy individuals with different *IL-1RL1* haplotypes were isolated and differentiated into Th2 cells followed by CD3/CD28 activation with or without exogenous interleukin (IL)-33. RNA sequencing of Th2 cells was used to generate a gene signature, which was then validated in various datasets (CD4+, Human Lung Cell Atlas (HLCA), INDURIAN, U-BIOPRED) for clinical asthma phenotype associations. b) Clinical characteristics of the peripheral blood mononuclear cell donors selected from NORM (controls) and ROORDA (asthma patients) cohorts. Data are presented as mean±sd or median (interquartile range), unless otherwise indicated. c and d) Volcano plots showing gene expression changes in Th2 cells (n=29), after CD3/CD28 activation (c) and by the additional presence of IL-33 (d), with 12 038 genes tested. Significant gene upregulation (brown dots) and downregulation (orange dots) are highlighted. False discovery rate-adjusted p-value threshold is 0.05. e–h) Volcano plots for differentially expressed genes induced by IL-33 in high risk (n=14; e) and low risk haplotype carriers (n=15; f), or in asthma patients (n=15; g), and healthy controls (n=14; h). i–k) Enrichment of the Th2 IL33 gene signature in the HLCA [7], in CD4+ T cells isolated from bronchial biopsies of patients with asthma and healthy controls [8], and in bronchial biopsies from patients with asthma and healthy controls from the INDURAIN cohort [9]. l and m) Enrichment scores for the IL-33 gene signature in U-BIOPRED sputum transcriptomic data, analysed across different disease groups, asthma phenotypes, and transcriptome-associated clusters (TAC) groups generated through unsupervised clustering of transcriptome data [10]. DC: dendritic cell; NK: natural killer; GSVA: gene set variation analysis; MMA: mild/moderate asthma patients; SA: severe asthma patients. Statistical tests include t-test and Wilcoxon rank-sum test.

**FIGURE 1:**
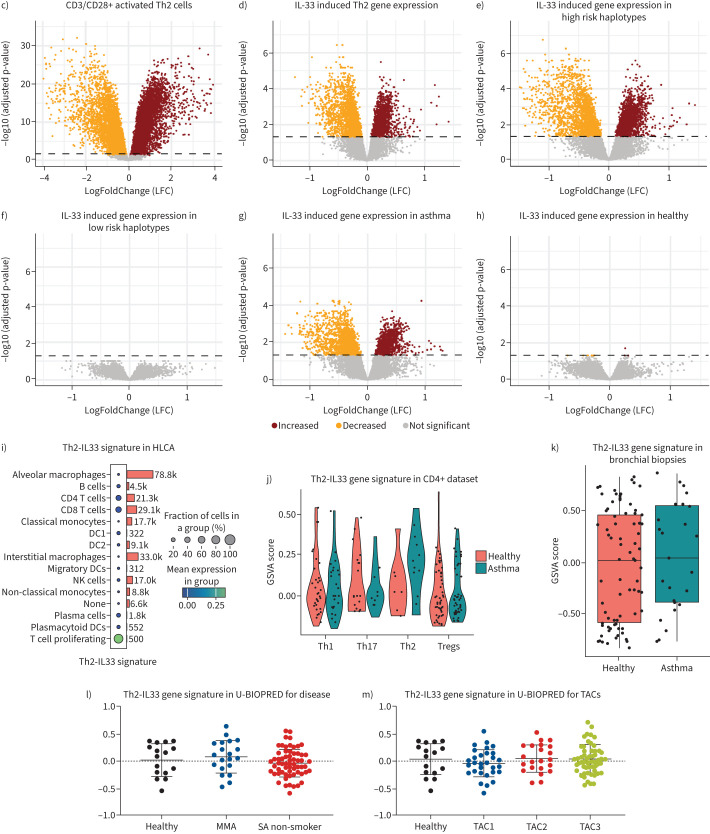
Continued.

CD3/CD28 restimulation of Th2 cells resulted in 9299 significantly differentially expressed genes compared to unstimulated Th2 cells (figure 1c), mapping to pathways related to T cell receptor signalling, proliferation and cytokine production (not shown). No differences were observed between *IL-1RL1* haplotype groups or disease categories for this response (not shown). Presence of IL-33 during CD3/CD28 restimulation resulted in 3677 DEGs, using *IL-1RL1* haplotype and disease status as covariates (figure 1d). Stratified analyses per haplotype group revealed that Th2 cells with the *IL-1RL1* risk haplotype exhibited significant transcriptional changes (3715 DEGs) upon IL-33 exposure, in strong contrast to cells with the low risk haplotype (0 DEGs; figure 1e and f). Disease group stratification revealed that in Th2 cells from asthma patients, presence of IL-33 resulted in 2524 DEGs, compared to only 10 DEGs in Th2 cells from healthy controls (figure 1g and h). IL-33-induced genes overlapped completely between asthma patients and *IL-1RL1* risk haplotype carriers, with 1299 (nearly 35%) DEGs reaching genome-wide significance only in the *IL-1RL1* risk haplotype group. We did not observe an effect of *IL-1RL1* haplotype on the level of IL-1RL1a RNA or protein. Taken together, these findings highlight the combined impact of *IL-1RL1* haplotype and disease status on the sensitivity of Th2 cells to IL-33, suggesting that the most significant effects of IL-33 in Th2 cells were observed in asthma patients with the *IL-1RL1* high-risk haplotype.

Next, we explored whether the Th2-specific IL-33-induced gene signature could be used as a transcriptional biomarker to identify a subgroup of asthma patients with high activity of the IL-33 pathway. To achieve this, we derived a T cell-specific IL-33 response signature by selecting the strongest IL-33 induced genes that are specifically expressed in T cells based on the HLCA [7], a list of 20 genes. We next tested enrichment of this 20-gene signature in transcriptomic datasets from lung tissue samples of patients with asthma and healthy controls. Analysis in the HLCA confirmed high expression in CD4, CD8 and proliferating T cells, underscoring the cell-type specificity of the signature (figure 1i). Using our previously published dataset of tissue-resident Th cells from bronchial biopsies [8], we show that the 20-gene signature is highly expressed in airway wall-resident Th2 cells from patients with asthma (figure 1j), but not in those from healthy controls or in other effector Th cell subsets (Th1, Th17 or Tregs), confirming specificity of the signature for pathologic Th2 cells in asthma. Further evaluation of the gene signature in bulk RNA-sequencing data from asthma patients and controls of the INDURAIN cohort [9] revealed no significant enrichment in bronchial biopsies of asthma patients relative to healthy controls (figure 1k). Since the gene signature was generated in effector Th2 cells, we next assessed enrichment of the gene signature in sputum transcriptomic data from patients with severe asthma from U-BIOPRED [10]. However, the signature did not show any significant enrichment across disease groups or transcriptome-associated cluster molecular phenotypes in the U-BIOPRED data. (figure 1l and m) [10].

In conclusion, our results show that IL-33 has a major impact on gene expression in activated Th2 cells, which is dependent on disease status and *IL-1RL1* haplotype: sensitivity of Th2 cells for IL-33 is highest in patients with asthma that carry the *IL-1RL1* risk haplotype. A T cell-specific IL-33 response signature is enriched in airway wall-resident Th2 cells in patients with asthma, underscoring its specificity. However, we also show that it does not detect activity of the IL-33 pathway in bulk transcriptomic datasets from patients with asthma, likely due to the rarity of effector Th2 cells in tissue samples. Moreover, we also realise that, although we carefully tried to match the disease and haplotype groups on clinical parameters, there were fewer males in the healthy group, which may act as a potential confounder. Therefore, to allow identification of patients with increased activity of the IL-33 pathway, other approaches need to be considered, such as transcriptomic signatures in more prevalent IL-33 responsive cell types such as macrophages, or the use of a combination of genetic and/or epigenetic features [11]. In this respect, it is of interest to note that our efforts to establish an IL-33 response signature in bronchial epithelial cells, the main cell type in a brush or biopsy form the lower airways, failed to identify any IL-33 induced genes [12]. This underscores the continued need for the development of better prognostic biomarkers of IL-33 pathway activity that can predict the treatment response to interventions targeting the pathway and that could be used for patient stratification in precision medicine for asthma or COPD.

## Shareable PDF

10.1183/13993003.00005-2024.Shareable1 This one-page PDF can be shared freely online.
Shareable PDF ERJ-00005-2024.Shareable

## Data Availability

Further data relating to this work are available from https://discovair.org/data-sets.

## References

[1] Porsbjerg C, Melén E, Lehtimäki L, et al. Asthma. Lancet 2023; 401: 858–873. doi:10.1016/S0140-6736(22)02125-0 36682372

[2] Portelli MA, Nicole Dijk F, Ketelaar ME, et al. Phenotypic and functional translation of IL1RL1 locus polymorphisms in lung tissue and asthmatic airway epithelium. JCI Insight 2020; 147: 144–157. 10.1172/jci.insight.132446 PMC7205441 32324168

[3] Cayrol C, Girard J-P. Interleukin-33 (IL-33): a nuclear cytokine from the IL-1 family. Immunol Rev 2018; 281: 154–168. doi:10.1111/imr.12619 29247993

[4] Saikumar Jayalatha AK, Hesse L, Ketelaar ME, et al. The central role of IL-33/IL-1RL1 pathway in asthma: from pathogenesis to intervention. Pharmacol Ther 2021; 225: 107847. doi:10.1016/j.pharmthera.2021.107847 33819560

[5] Cayrol C, Girard JP. IL-33: An alarmin cytokine with crucial roles in innate immunity, inflammation and allergy. Curr Opin Immunol 2014; 31: 31–37. doi:10.1016/j.coi.2014.09.004 25278425

[6] Ketelaar ME, Portelli MA, Dijk FN, et al. Phenotypic and functional translation of IL33 genetics in asthma. J Allergy Clin Immunol 2021; 147: 144–157. doi:10.1016/j.jaci.2020.04.051 32442646

[7] Sikkema L, Ramírez-Suástegui C, Strobl DC, et al. An integrated cell atlas of the lung in health and disease. Nat Med 2023; 29: 1563–1577. doi:10.1038/s41591-023-02327-2 37291214 PMC10287567

[8] Vieira Braga FA, Kar G, Berg M, et al. A cellular census of human lungs identifies novel cell states in health and in asthma. Nat Med 2019; 25: 1153–1163. doi:10.1038/s41591-019-0468-5 31209336

[9] Vermeulen CJ, Xu CJ, Vonk JM, et al. Differential DNA methylation in bronchial biopsies between persistent asthma and asthma in remission. Eur Respir J 2020; 55: 1901280. doi:10.1183/13993003.01280-2019 31699840

[10] Kuo CHS, Pavlidis S, Loza M, et al. T-helper cell type 2 (Th2) and non-Th2 molecular phenotypes of asthma using sputum transcriptomics in U-BIOPRED. Eur Respir J 2017; 49: 1602135. doi:10.1183/13993003.02135-2016 28179442

[11] Seumois G, Ramírez-Suástegui C, Schmiedel BJ, et al. Single-cell transcriptomic analysis of allergen-specific T cells in allergy and asthma. Sci Immunol 2020; 5: eaba6087. doi:10.1126/sciimmunol.aba6087 32532832 PMC7372639

[12] Saikumar Jayalatha AK, Jonker MR, Carpaij OA. Lack of a transcriptional response of primary bronchial epithelial cells from asthma patients and controls to IL-33. Am J Physiol Lung Cell Mol Physiol 2024; 326: L65–L70. doi:10.1152/ajplung.00298.2023 38050688

