# Supplementary material for: IL-33 induced gene expression in activated Th2 effector cells is dependent on IL-1RL1 haplotype and asthma status
Source: Eur Respir J. 2024 Jun 20;63(6):2400005. doi: 10.1183/13993003.00005-2024 (PMC11187316; doi:10.1183/13993003.00005-2024)
Supplement: Supplementary file 1 [file ERJ-00005-2024.Shareable.pdf]

SHAREABLE PDF

IL-33 induced gene expression in activated Th2 effector cells is dependent on IL-1RL1 haplotype and asthma status

Akshaya Keerthi Saikumar Jayalatha [1,8], Marlies E. Ketelaar[1,2,8], Laura Hesse[1,8], Yusef E. Badi[3], Nazanin Zounemat-Kermani[3], Sharon Brouwer[1], Nicole F. Dijk[2], Maarten van den Berge[4], Victor Guryev [5], Ian Sayers [6], Judith E. Vonk [7], Ian M. Adcock [3], Gerard H. Koppelman [2] and Martijn C. Nawijn [1]

[1]University of Groningen, University Medical Center Groningen, GRIAC Research Institute, Department of Pathology and Medical Biology, Groningen, The Netherlands. [2]University of Groningen, University Medical Center Groningen, GRIAC Research Institute, Beatrix Children's Hospital, Department of Pediatric Pulmonology and Pediatric Allergology, Groningen, The Netherlands. [3]National Heart and Lung Institute, Department of Respiratory Cell and Molecular Biology, Imperial College London, London, UK. [4]University of Groningen, University Medical Center Groningen, GRIAC Research Institute, Department of Pulmonary Diseases, Groningen, The Netherlands. [5]University of Groningen, GRIAC Research Institute and European Research Institute for the Biology of Ageing, Groningen, The Netherlands. [6]Centre for Respiratory Research, NIHR Biomedical Research Centre, School of Medicine, Biodiscovery Institute, University of Nottingham, Nottingham, UK. [7]University of Groningen, University Medical Center Groningen, GRIAC Research institute, Department of Epidemiology, Groningen, The Netherlands. [8]Shared first authorship.

Corresponding author: Martijn C. Nawijn (m.c.nawijn@umcg.nl)

Shareable abstract (@ERSpublications)
IL-33 response in Th2 cells is specific to asthma and represents a high risk haplotype, highlighting its role in airway wall cells. Yet, its detection is challenging in bulk asthma transcriptomes due to the scarcity of effector Th2 cells. https://bit.ly/3WhuMbo

Cite this article as: Saikumar Jayalatha AK, Ketelaar ME, Hesse L, *et al*. IL-33 induced gene expression in activated Th2 effector cells is dependent on IL-1RL1 haplotype and asthma status. *Eur Respir J* 2024; 63: 2400005 [DOI: 10.1183/13993003.00005-2024].

This extracted version can be shared freely online.

Copyright ©The authors 2024.

This version is distributed under the terms of the Creative Commons Attribution Licence 4.0.

Received: 17 June 2023
Accepted: 22 April 2024

To the Editor:

Asthma is a heterogeneous respiratory disease caused by the interaction between environmental and genetic factors [1]. The *IL-33* and *IL-1RL1* genes are strongly associated with childhood-onset and type-2 high asthma, and the asthma risk alleles amplify interleukin (IL)-33 pathway activity [2]. Environmental factors, such as allergens and viral infections, trigger bronchial epithelial cells to release IL-33, which can activate signalling by binding to the IL-1RL1/IL-1RAcP receptor complex [3], and contribute to hyper-responsiveness, remodelling and chronic type 2 inflammation of the airways [4]. *IL-1RL1* is expressed in immune and structural cells of the airways, such as epithelial cells, mast cells, macrophages, Th2 cells and type 2 innate lymphoid cells. *IL-1RL1* encodes two protein isoforms: the transmembrane receptor subunit (IL-1RL1b) and a soluble (IL-1RL1a) isoform that functions as an antagonistic decoy receptor. Human Th2 cells respond to IL-33 by enhancing cytokine production [5]. Genetic variation at the *IL-1RL1* locus, particularly rs1420101 in intron 5 and a block of four non-synonymous single nucleotide polymorphisms (SNPs) in full linkage disequilibrium in exon 11, alter *IL-1RL1* expression levels and IL-33 induced signalling activity [2, 6]. However, it is not known whether genetic variation at the *IL-1RL1* locus affects the response of Th2 cells to IL-33. Therefore, we tested whether *IL-1RL1* haplotype altered the IL-33 induced response of Th2 cells from healthy controls and patients with asthma. Moreover, we explored whether IL-33-induced gene signatures from Th2 cells could identify subgroups of asthma patients in transcriptomic datasets.
